# Pain and Opioid use Outcomes Following Minimally Invasive Sacroiliac Joint Fusion with Decortication and Bone Grafting: The Evolusion Clinical Trial

**DOI:** 10.2174/1874325001711011440

**Published:** 2017-12-27

**Authors:** Ali Araghi, Robert Woodruff, Kyle Colle, Christopher Boone, Lisa Ingham, Antoine Tomeh, Louis C Fielding

**Affiliations:** 1 The CORE Institute, Phoenix, Arizona, United States; 2 Black Hills Orthopedic and Spine Center, Rapid City, South Dakota, United States; 3 Regional Brain and Spine, Cape Girardeau, Missouri, United States; 4 Bellevue Bone and Joint Physicians, Bellevue, Washington, United States; 5 Zyga Technology, Inc. Minnetonka, Minnesota, United States; 6 Northwest Orthopaedic Specialists, Spokane, Washington, United States; 7 Tahoe Labs, LLC, 1057 Montgomery St., San Carlos, 94070 California, United States

**Keywords:** Sacroiliac Joint Dysfunction,, Sacroiliac Joint Fusion,, SI Joint,, SI Joint Arthrodesis,, Minimally Invasive Surgery,, Sacroiliac,, Fusion,, Arthrodesis.

## Abstract

**Purpose:**

This report documents six-month results of the first 50 patients treated in a prospective, multi-center study of a minimally invasive (MI) sacroiliac (SI) joint fusion system.

**Patients and Methods:**

This cohort includes 50 patients who had MI SI joint fusion surgery and completed 6 month follow-up. Average age at baseline was 61.5, 58% were female, and SI joint-related pain duration was ≥2yrs in 54.0% of patients. Visual Analog Scale (VAS) SI joint pain, Oswestry Disability Index (ODI), quality of life and opioid use were assessed preoperatively and at 6 months.

**Results:**

At 6 months, mean VAS pain demonstrated a significant reduction from 76.2 at baseline to 35.1 (54% reduction, p<0.0001), with 72% of patients attaining the minimal clinically important difference (MCID, ≥20 point improvement). Mean ODI improved from 55.5 to 35.3 at 6 months (p < 0.001), with 56% of patients achieving the MCID (≥15 point improvement). Prior to surgery 33/50 (66%) of patients were taking opioids, but by 6 months the number of patients taking opioids had decreased by 55% to 15/50 (30%). Few procedural complications were reported. Two procedure-related events required hospitalization: a revision procedure (2%) for nerve impingement and one case of ongoing low back pain.

**Conclusion:**

Analysis of patients treated with MI SI joint fusion using the SImmetry System demonstrated that the procedure can be performed safely and results in significant improvements in pain, disability, and opioid use at 6 months. Longer term follow-up in this study will determine whether these improvements are durable, as well as the associated radiographic fusion rates.

## 1 INTRODUCTION

Minimally invasive (MI) sacroiliac (SI) joint fusion has become a preferred treatment alternative for pain originating in the SI joint that is refractory to conservative treatment [1-3]. With the increasing popularity of this procedure, there are now more than 20 implantable devices indicated for SI joint fixation, yet there is minimal published clinical data to understand the merits or comparative effectiveness of the different systems that are available. Within the clinical publications of MI SI joint fusion, there is even less data regarding actual fusion rates [4].

An MI SI joint fusion system incorporating decortication, bone grafting and fixation with threaded implants has demonstrated encouraging results in smaller case series [5, 6]. This study is an interim report of the six month results of the first 50 patients enrolled in a prospective, multi-center study of this system that will evaluate pain, quality of life, opioid use and radiographic fusion outcomes.

## 2 METHODS

### 2.1 Trial Design

This is a report of the first 50 patients participating in a prospective, multicenter evaluation of the SImmetry Sacroiliac Joint Fusion System (Zyga Technology, Minnetonka, MN) in the EVoluSIon study (EVSI). EVSI is to be conducted at up to 40 centers in the United States, enrolling up to 250 patients, and is registered on clinicaltrials.gov as NCT02074761. The protocol was approved by each participating center’s institutional review board and written informed consent was obtained from all patients prior to enrollment. Patients were screened from each investigator’s population of patients indicated for minimally invasive SI joint fusion. The key inclusion criteria for participation were 18 years of age or greater; at least six months of non-operative management of SI joint pain; three positive provocative tests (FABER, Gaenslen, Compression, Thigh Thrust, Distraction) [7]; at least one positive diagnostic SI joint injection [8, 9]; Visual Analog Scale (VAS) SI joint pain score of 60 or greater; and Oswestry Disability Index (ODI) score of at least 40. Potential participants were excluded for any of the following reasons: pelvic soft tissue or bony tumors; trauma causing fracture of the sacrum or iliac bones or spinal trauma leading to a neurological deficit; history of a central nervous system disorder(s); painful hip and/or knee arthrosis with potential progression to hip and/or knee arthroplasty; indication for or awaiting other spine surgery; pregnancy or planned pregnancy in the next two years; uncontrolled insulin dependent diabetes mellitus; chemical dependency or substance abuse; receiving or seeking worker’s compensation, disability remuneration, or involved in litigation related to low back or SI joint pain; and history of significant emotional or psychosocial disturbance.

Baseline data collection included medical history, prior treatments and surgeries, provocative maneuvers to confirm the diagnosis of SI joint dysfunction, diagnostic SI joint injections and pain medication usage. Eligible patients were asked to complete a questionnaire which included self-evaluation of various elements: 1) SI joint pain as measured by a VAS from 0 to 100, where 0 represented no pain and 100 represented the worst possible pain; 2) disability due to low back pain as measured by the validated ODI survey; and 3) quality of life through the EQ-5D and SF-36 questionnaires. The patient questionnaires and pain medication usage were collected pre-operatively and at specified time points of 6 weeks, 3 months, 6 months, and will be continued through 12 months, and 24 months postoperatively. Patient questionnaires were self-reported and completed by each patient prior to meeting with the investigator to limit bias. All patient qualification and endpoint data were monitored and source verified for accuracy in reporting and compliance. Data were collected and reported via electronic database (Fortress Medical Systems, Hopkins, MN).

Fusion will be evaluated through collection of thin-slice computed tomography (CT) images at 12 months. Fusion assessments for the primary endpoint will be analyzed and reported after at least 50 patients have completed the 12 month visit.

### 2.2 Surgical Procedure

The surgical procedure included MI SI joint fusion using the SImmetry System in accordance with approved labeling. The implant system includes a 12.5 mm diameter cannulated implant placed through the location of decortication and an 8.5 mm diameter anti-rotational implant for mechanical stability. Both are threaded implants made of titanium with a surface roughness designed for osseointegration.

**Fig. (1):**
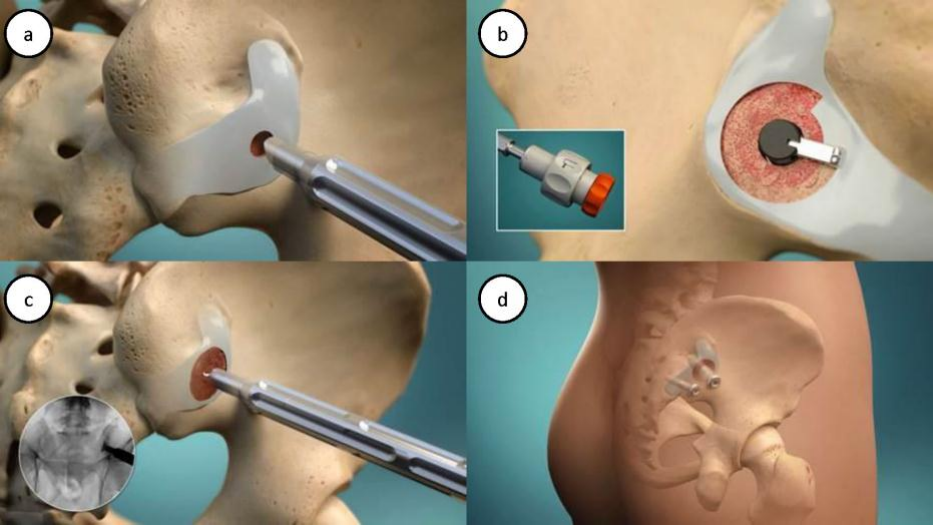
(a) Minimally invasive lateral access of the SI joint; (b) Decortication of the joint (inset: user-interface of decorticator instrument); (c) Delivery of bone graft into the actively bleeding fusion bed (inset: fluoroscopic image during decortication); (d) Final placement of the threaded implant through the area of decortication and a secondary device for stability.

The procedure has been described elsewhere [10-12]. Briefly, four steps are performed as in (Fig. **1**): minimally invasive lateral access; joint preparation via decortication; bone graft placement; and implant delivery. The joint preparation occurs through use of a proprietary decorticator to remove cartilage and prepare up to 50% of the SI joint surface as an active bleeding fusion bed for bone graft material. Bone graft (autologous bone with or without allograft and/or demineralized bone matrix) is then packed into the decorticated area, before the cannulated implant is placed across the area of decortication. A secondary anti-rotational implant is then placed.

### 2.3 Statistical Methods

The primary objectives of the broader trial, of which these patients represent an initial series, are to evaluate SI joint pain relief and radiographic evidence of fusion after implantation with the SImmetry System. Ultimately two primary endpoints will be studied which include: 1) A decrease in SI joint pain from baseline to 6 month follow-up as evaluated by use of a patient-reported 100mm VAS; and 2) Radiographic evidence of SI joint fusion at 12 and 24 months post-surgery. Fusion is defined as presence of a continuous segment of solid bridging bone that extends from the sacrum to the ilium. Predefined statistical analyses described in the protocol include the primary endpoint analysis of VAS SI joint pain scores beginning with the 6-month follow-up visit. Pain relief post-implantation was evaluated at each follow-up interval with descriptive statistical analyses (mean, standard deviation, median, minimum and maximum). Interim analyses to monitor the progress of the trial are predefined at intervals of every 50 enrolled patients at 6 months, 12 months and 24 months.

VAS pain and ODI reductions were also defined in terms of minimal clinically important difference (MCID) of 20 points on the VAS scale and 15 points on the ODI scale. Multiple secondary analyses were predefined in the protocol, including assessment of ODI, EQ-5D, SF-36, procedure data, employment status, safety, and comparison of baseline and procedural data to outcomes of pain reduction and fusion. Statistical analyses to assess improvements following treatment using the pre-operative and follow-up VAS, ODI, and quality of life measures for each patient over time, using linear repeated measures models fit via maximum likelihood and a compound symmetric covariance structure, with results presented by visit and for the overall follow-up period. 95% confidence intervals on the mean changes from the pre-operative result in ODI and VAS scores were computed. Procedural data were summarized with descriptive statistics and 95% confidence intervals. Subgroup analyses for the primary endpoints were planned to determine if baseline or procedural characteristics differentiate subjects in terms of outcomes. Logistic and linear regression models were used for these analyses for the endpoints as appropriate.

While 12 month follow-up results were not available at this interim analysis, the statistical analysis plan required that rates for fusion be reported as percentages of implanted subjects who show evidence of radiographic fusion at the 12 and 24 month follow-up visits.

## 3 RESULTS

### 3.1 Patient Demographics and Baseline Characteristics

The analysis cohort presented here includes the first 50 patients enrolled who had MI SI joint fusion surgery and completed a 6 month follow-up visit. This was accomplished by enrolling patients at 13 institutions. The average age at baseline was 61.5 years, the majority were female (58.0%), and SI joint-related pain duration was at least two years in 54.0% of patients. Half of the patients never used tobacco and only four patients (8.0%) were currently using cigarettes at the time of enrollment. The vast majority of patients were not working pre-surgery (82%) and many had a prior lumbar surgery (54%).

The mean SI joint VAS pain was 76.2±11.3 (mean±SD; range: 60-100) and the ODI was 55.5 ±14.4 (range 20-80; ODI baseline score below 40 is a protocol deviation as the baseline requirement for inclusion was at least 40). Sixty-six percent (66%) of patients were taking opioids prior to surgery. Baseline characteristics are further summarized in Table **1**.

### 3.2 Surgical Procedure

Fifty (50) patients underwent planned SI joint fusion surgery with the SImmetry System per the manufacturer’s instructions for use. All but one procedure (49) were unilateral; the other procedure (1) was bilateral. The average procedure time was 57 minutes (range 25 to 92 minutes) and estimated blood loss averaged 69cc. The hospital stay duration was a mean of one day with 96.0% of patients discharged within less than 2 days of the procedure. Procedural data are summarized in Table **2**.

### 3.3 Outcomes

At the 6 month follow-up visit, mean VAS SI joint pain had decreased more than 41 points, from 76.2 at baseline to 35.1. This represented a statistically significant (p < 0.0001) reduction in pain of 54%. The proportion of patients attaining the MCID (**i.e**., a reduction of at least 20 out of 100 points) was 72% (N=36). For patients achieving the MCID, their average reduction in pain during the same time frame was 74%.

Similar to the reduction in pain, ODI results demonstrated a significant improvement in disability scores after SI joint fusion surgery. Mean ODI improved from 55.5 at baseline to 35.3 at the 6 month follow-up (p < 0.001), demonstrating a statistically significant and clinically meaningful improvement in disability related to SI joint pain. The MCID of a decrease of 15 points was achieved by 56% (N=28) of patients. Covariate analysis of VAS and ODI outcomes with baseline and procedural characteristics did not demonstrate any statistically significant findings during this analysis.

In addition to improvements in pain and disability, a significant decline in opioid use was reported. Prior to minimally invasive surgery 33/50 (66%) of patients were taking opioids, but by 6 months post-surgery the number of patients taking opioids had decreased to 15/50 (30%, p=0.0004). Additional evaluation of patients reporting a MCID in pain showed only 19% (7/36) of these patients continuing to use opioids 6 months postoperatively. A decrease in non-opioid pain medications was reported as well. At baseline 70.0% of patients were taking NSAIDs, 46.0% were taking other analgesics, and 22.0% were taking steroids. The percentage of patients taking NSAIDS, analgesics and steroids at the 6 month follow-up decreased to 20.4%, 22.5% and 2.0%, respectively.

Quality of life assessments were collected during follow-up visits using both the EQ-5D and SF-36 questionnaires. The mean EQ-5D time trade-off index was 0.51 at baseline and increased to 0.69 (p<0.0001) 6 months post-surgery, a statistically significant change. In the same assessment, the global health thermometer rating was 56.1 at baseline compared to 68.2 (p<0.0001) 6 months post-surgery.

The SF-36 includes both physical and mental components of a norm-based quality of life questionnaire. The mean physical component was 30.84 at baseline and increased to 36.41 (SD 10.52) at the 6 month time point (p=0.0001). Meanwhile the mental component changed from a mean baseline score of 42.21 to 48.50 (SD 11.34) at 6 months (p=0.0016). Both represent statistically significant improvements in this cohort.

### 3.4 Complications

Few complications related to the procedure or implanted devices were reported. Serious adverse events, including events resulting in hospitalization, were reported for two patients. One patient had radiculopathy post-surgery and nerve impingement was suggested by CT as the implanted device extended into the S1 canal. This event led to a revision procedure in which the implant was replaced with a shorter length device. Radiculopathy symptoms resolved immediately after the revision procedure. The second serious event was an ongoing case of low back pain resulting in inpatient hospitalization to manage pain.

Investigators reported eight other surgical related events, but none were deemed serious in nature, nor required intervention. There was one revision (2%) in 50 patients through the 6 month visit as noted above.

## 4 DISCUSSION

Early results from this prospective, multicenter trial demonstrated that MI SI joint fusion surgery with decortication, bone grafting and fixation with threaded implants resulted in an average pain reduction of 54%, and that 72% of patients had a MCID (improvement >20 points) within the first 6 months post-surgery. Improvement in pain reduction was echoed in the results for disability where a statistically significant and clinically substantial improvement was reported by patients. Residual pain may be due to multifactorial degenerative lumbosacral pathology, neuroplastic effects or other inorganic causes, however was characteristic of this patient population [13, 14]. The most significant finding from this interim analysis, however, was the reduction in opioid use. More than half of the patients taking opioids at baseline were no longer taking those medications at the 6 month follow-up; only 30% of subjects were still using opioids for pain management at 6 months. In comparison, other clinical studies of MI SI joint fusion reported 58% of patients still using opioids at 6 months and 48% to 55% of patients still using opioids at 24 months postoperatively [13, 14]. Similar reductions were seen in non-opioid pain medication use, including NSAIDs, analgesics and steroids, further indicating that these patients experienced substantial relief of pain 6 months postoperatively.

SImmetry is one of numerous products cleared by the Food and Drug Administration for minimally invasive implantation in patients with SI joint dysfunction. These devices are neither experimental nor investigational. While clinical trial data are not required, only a few of the 20+ implantable devices indicated for SI joint fixation have published clinical data available for comparison with the current trial. The most relevant information is from a two-year published trial with triangular titanium implants, called SIFI [13, 15]. SIFI was sponsored by the device manufacturer, had similar inclusion and exclusion criteria, and a single arm enrollment design of 172 patients followed through two years (Table **3**). The study population only differed in the baseline lower limits for VAS and ODI, such that SIFI enrolled patients with a minimum VAS of 50 and ODI of 30, while the current study enrolled only those with a minimum VAS of 60 and ODI of 40. Both studies are single arm, prospective, multi-center trials with a primary objective of pain reduction at 6 months, with similar data collection and follow-up intervals. The current EVSI trial has an additional primary endpoint of fusion which will be radiographically assessed by an independent core laboratory at 12 and conditionally 24 months if fusion is not yet demonstrated at 12 months. The products used in each trial have a similar indication and both implore a minimally invasive technique, which allow for a device-to-device comparison of the trial results.

EVSI and SIFI had similar patient populations with the primary differences being average age (EVSI=61.5, SIFI 50.9), gender (EVSI=58.0% female; SIFI=69.8% female), baseline opioid use (EVSI=66%, SIFI=76%), and sample size (EVSI=50, SIFI=172). Baseline VAS (EVSI=76.2, SIFI=79.8) and ODI (EVSI=55.5, SIFI=55.2) were very similar despite the differences in age, gender, opioid use and inclusion criteria. Revisions occurred in 2.0% of patients in EVSI through 6 months and 4.7% of patients in SIFI through two years. In comparing 6 month outcomes of these two trials, both achieved a statistically significant reduction in average pain compared to pre-surgery (54% EVSI, 62% SIFI), and a reduction in disability (20 points EVSI, 23 points SIFI). Both trials showed a reduction in opioid use through 6 months as well, although the EVSI cohort had a much greater reduction (33/50 to 15/50 or 55% reduction in EVSI; 76% to 60%, or 21% reduction in SIFI) [13, 15].

The similarity in results of the EVSI and SIFI studies demonstrate that fixation of the SI joint results in pain relief, regardless of the type of implant used. It is notable that fusion rates with MI SI joint fusion systems are not well reported in the literature [4], in contrast to other studies of spinal fusion where radiographic arthrodesis is considered an important success criterion [16, 17]. Long-term evidence of whether SI joint fusion is achieved through fixation still needs to be evaluated, and is a key objective of this study. Additionally, factors that contribute to the long-term pain relief of patients should continue to be studied. Additional patients and longer-term follow-up in the EVSI trial may help to define similarities or further distinctions between different types of implants for the SI joint.

Previous prospective studies have demonstrated the importance of fixation of the SI joint in reducing pain and improving disability status. Comparatively, reduction in VAS pain 6 months after MI SI joint surgery has been reported as 63% and 50% with patient sample sizes of 101 and 18, respectively [12, 14]. These are comparable to the EVSI cohort of 50 patients in which a 54% reduction in average pain was shown during the same interval.

The greatest limitations with this trial are the sample size and limited follow-up. The current data represent the first interim analysis of what will be the largest cohort of patients prospectively enrolled in a trial to evaluate both fusion and pain following MI SI joint fusion surgery. An additional 200 patients are planned to be enrolled to provide enough statistical power to determine contributing factors to fusion and pain relief. At present, this interim analysis of 50 patients provides sufficient positive outcome data to validate continuing with the trial protocol through two years of follow-up.

Another limitation of this trial is a lack of a control group. The comparison of minimally invasive SI joint fixation to non-surgical therapy was previously established in a randomized trial reported by Polly, *et al*. [14] Results from the trial demonstrated that the surgery group had a substantially significant improvement in pain compared to non-surgical therapy group. Greater improvement in disability and quality of life was also shown in the surgical group with results lasting through two years. In light of the superior results shown with SI joint surgery, we feel there is no clinical equipoise to suggest that additional randomized controlled trials would be acceptable [18]. Instead, the purpose of this trial is to evaluate patient outcomes using the technology of decortication, bone grafting and threaded implants described herein. In this discussion the results are compared to a similar study of another MI SIJ fusion system. While this comparison provides relevant context, it must be acknowledged that differences in methodology, investigational site standards of care, and even changes in public attitudes toward opioid painkillers could impact differences seen in the results of the two studies.

This trial evaluated a 6 month endpoint for pain reduction, but clinically the ultimate goal is long-term pain relief. While implantation of SI joint devices aims to stabilize the joint, the ultimate goal is fusion of the joint to achieve long-term relief. Fusion of the SI joint has been understudied and reports of SI joint fusion range from 6 months to five years [4, 19-21]. Additional follow-up and evaluation of radiographs collected at 12 and 24 months will help to further define permanent fusion of the SI joint and patient and procedural characteristics that may contribute to fusion and long-term pain relief.

## CONCLUSION

Minimally invasive SI joint fusion surgery with decortication, bone graft and threaded implant fixation results in pain and disability improvement through 6 months with few complications. This interim analysis demonstrates that the procedure can be performed safely, resulting in an average reduction in pain of 54%, significantly improved disability, and a substantial reduction in the use of opioids of which current use is considered a national crisis. While pain and disability improvement is comparable to similar studies, the reduction in opioid use (55%) was much greater than previously reported in other studies. Additional pain and fusion data from more patients and longer follow-up will continue to further delineate clinical and radiographic outcomes.

## Figures and Tables

**Table 1:** Baseline Characteristics of Trial Participants.

**Demographic**	**-**	**N=50**
Age, mean ± SD (range)	**-**	61.5 ± 13.7 (21.7, 85.1)
Female, n (%)	**-**	29 (58.0%)
BMI, mean ± SD (range)	**-**	30.1 ± 7.1 (17.7, 50.2)
Tobacco history, n (%)	Never used	25 (50.0%)
	Current smoker	4 (8.0%)
	Past smoker	21 (42.0%)
Work status, n/N (%)	Full-time	8 (16.0%)
	Part-time due to SI joint pain	1 (2.0%)
	Part-time voluntarily	0 (0%)
	Short term disability	1 (2.0%)
	Long term disability	10 (20.0%)
	Not working voluntarily	7 (14.0%)
	Unemployed	23 (46.0%)
Duration of SI joint pain symptoms, n/N (%)	6 mo - 1 yr	13 (26.0%)
	1 - 2 yr	10 (20.0%)
	> 2 yr	27 (54.0%)
Taking opioids, n/N (%)		33 (66%)
Prior SI joint treatment, n/N (%)	Physical therapy	43 (86.0%)
	Chiropractic care	14 (28.0%)
	Ablation	5 (10.0%)
Prior spine history, n/N (%)	Lumbar fusion	14 (28.0%)
	Lumbar stenosis	8 (16.0%)
	Lumbar decompression	5 (10.0%)
	Degenerative disc disease	28 (56.0%)
	Bilateral SI joint disease	17 (34.0%)
	Spondylolisthesis	11 (22.0%)
	Osteoarthritis	10 (20.0%)
VAS pain, mean ± SD (range)	**-**	76.2 ± 11.3 (60, 100)
ODI, mean ± SD (range)	**-**	55.5 ± 14.4 (20, 80)
EQ-5D	Index value (TTO)	0.5 ± 0.1 (0.2, 0.8)
	VAS score	56.1 ± 23.8 (0, 99.0)
SF-36	Physical Component Summary	30.84 ± 6.82
	Mental component summary	42.21 ± 13.01

**Table 2:** Surgical procedure data. data summarized as mean ± SD (minimum, maximum) or n/N (%).

	**-**	**N=50**
Procedure duration (minutes)	**-**	57.4 ± 18.3 (25.0, 92.0)
Fluoroscopy time (minutes)	0-2 minutes	24 (48.0%)
	2-4 minutes	15 (30.0%)
	> 4 minutes	11 (22.0%)
Total contrast used (cc)	**-**	4.4 ± 15.1 (0.0, 100.0)
Estimated blood loss (cc)	0-50 cc	35 (70.0%)
	51-100 cc	8 (16.0%)
	> 100 cc	7 (14.0%)
Length of hospital stay (days) mean ± SD (range)	**-**	1.0 ± 0.6 (0.0, 3.0)
	Same day	7 (14.0%)
	1 day	36 (72.0%)
	2 days	5 (10.0%)
	> 2 days	2 (4.0%)

**Table 3:** Comparison of baseline and 6 month outcomes of similar trials with MI SI joint implants.

**Characteristic**	**EVSI** **N=50**	**SIFI** **N=172**
Age, mean years	61.5	50.9
Female Gender	58.0%	69.8%
VAS – Baseline, mean	76.2	79.8
VAS Reduction – 6 Months, mean	41.4 points	49.8 points
ODI – Baseline, mean	55.5	55.2
ODI Reduction – 6 Months, mean	20 points	23 points
Patients Using Opioids – Baseline	66%	76%
Patients Using Opioids – 6 Months	30%	60%
